# Perioperative Blood Transfusions and Anastomotic Leak After Colorectal Surgery for Cancer in an Australian Hospital

**DOI:** 10.1007/s12029-023-00947-y

**Published:** 2023-06-19

**Authors:** Fraser Hugh Simpson, Krish Kulendran, Stephanie Yerkovich, Andrew Beatty, David Flynn, Derek Mao, Taylor Brooks, Phoebe Wood, Manju D. Chandrasegaram

**Affiliations:** 1 Department of General Surgery, The Prince Charles Hospital, Brisbane, QLD Australia; 2 Northside Clinical School, School of Medicine, The University of Queensland, Brisbane, QLD Australia; 3 School of Clinical Medicine, The University of Queensland, Brisbane, Australia

**Keywords:** Colorectal cancer, Blood transfusion, Australia, Anastomotic leak, Primary anastomosis

## Abstract

**Purpose:**

Peri-operative blood transfusion has been identified as a risk factor for anastomotic leak in recent studies, but little is known about which patients are at risk for blood transfusion. This study aims to assess the relationship between blood transfusion and anastomotic leak and factors predisposing to leak in patients undergoing colorectal cancer surgery.

**Methods:**

This retrospective cohort study was conducted in a tertiary hospital in Brisbane, Australia, between 2010 and 2019. A total of 522 patients underwent resection of colorectal cancer with primary anastomosis with no covering stoma and the prevalence of anastomotic leak was compared between those who had had perioperative blood transfusion(s) and those who had not.

**Results:**

A total of 19 of 522 patients undergoing surgery for colorectal cancer had developed an anastomotic leak (3.64%). 11.3% of patients who had had a perioperative blood transfusion developed an anastomotic leak whereas 2.2% of patients who had not had a blood transfusion developed an anastomotic leak (*p* = 0.0002). Patients undergoing procedure on their right colon had proportionally more blood transfusions and this approached statistical significance (*p* = 0.06). Patients who received a greater quantity of units of blood transfusion prior to their diagnosis of anastomotic leak were more likely to develop an anastomotic leak (*p* = 0.001).

**Conclusion:**

Perioperative blood transfusions are associated with a significantly increased risk of an anastomotic leak following bowel resection with primary anastomosis for colorectal cancer.

## Introduction

Clinically significant anastomotic leak is one of the most dreaded complications in colorectal surgery. It is strongly associated with prolonged hospital stay [1], increased treatment related costs [2], and significant short- and long-term morbidity and mortality [3]. The incidence of anastomotic leak in colorectal surgery has been previously reported to be between 1.6 and 14% and mortality has been reported to be between 12 and 30% [4–7].

The etiology of anastomotic leak is multifactorial and relate to technical factors and patient factors. One factor that is thought to contribute to anastomotic leak is the interplay between systemic inflammation and immune regulation [8, 9]. Risk factors for anastomotic leak after colorectal surgery are male gender, high American Society of Anesthesiologists (ASA) grade, BMI greater than 30 kg/m^2^, and the operative urgency, with emergency operations having a higher leak rate than planned, elective surgery [10].

Anastomotic leaks have a significant impact on patient outcomes. They are associated with increased rates of local tumor [11]. In addition, the management of an anastomotic leak with either a temporary or definitive stoma bears a significant impact on patient’s quality of life [12].

Understanding the risk factors for an anastomotic leak may enable tailored mitigation of this risk for an individual patient depending on their risk profile [13]. Recent studies have shown an increased risk of anastomotic leak in patients following colorectal surgery who have had blood loss greater than 100mls or received a perioperative blood transfusion [14, 15]. These studies do not specifically look what risk factors predispose patients to receiving a peri-operative blood transfusion or individually examine the outcomes with left and right colon surgery.

In this study, we aim to see if perioperative blood transfusions increase the risk of anastomotic leak in patients who have restorative colorectal cancer surgery with a primary anastomosis. This study also aims to investigate risk factors for perioperative blood transfusion.

## Methods

### Study Design and Data Source

The Prince Charles Hospital (TPCH) in Brisbane, Australia, is a quaternary cardiothoracic hospital. TPCH provides care for colorectal cancer patients and maintains a detailed clinicopathological database on these patients. A retrospective study of colorectal cancer patients from 2010 to 2019 at TPCH was done to look at the association between receiving a perioperative blood transfusion and the development of an anastomotic leak. Patient charts were individually reviewed, and data was compiled by trained medical practitioners. Operation notes were reviewed for type of procedure and presence of anastomosis. Anesthetic and post-operative notes were reviewed to assess if a blood transfusion was given either during the procedure or afterwards. This study was approved by the institutional ethics board.

### Study Population

Patients who underwent colorectal cancer surgery at TPCH between January 2010 and December 2019 were included in the study. Patients were included if they had a lower gastrointestinal anastomosis following resection of their colorectal cancer. Patients were excluded if they had a covering stoma at time of initial procedure or if they had endoscopic resection of their malignant lesion alone. Patients were included if they had resection of a malignant lesion endoscopically that that underwent resection. Patients below the age of 12 were excluded from the study. Patients were excluded from the study if it was unclear if they received a blood transfusion.

### Measures and Definitions

Basic demographic data was collected on each patient including age, gender, height, weight, BMI, and American Society of Anesthesiologists (ASA) grade. Additional information on the patient’s comorbidities were collected.

For the purposes of this study, anastomotic leak was defined as leakage of gastrointestinal luminal contents from the site of the lower gastrointestinal anastomosis [16]. This was diagnosed radiographically or clinically based on radiographic evidence in CT scans ordered to evaluate for potential anastomotic leak, leakage of gastrointestinal contents into surgical drains or intraoperative evidence of anastomotic leak. Perioperative blood transfusion was defined as a blood transfusion of packed red blood cells between fourteen days prior to the procedure or up to seven days after the procedure. In the case of patients who were diagnosed with a clinically significant anastomotic leak, blood transfusions were only counted if they happened before the diagnosis of the leak.

### Statistics

The null hypothesis was that there was no difference in anastomotic leak between the perioperative transfusion group and the no perioperative transfusion group. Continuous variables are reported as median and range. Statical analysis was performed with Fischer’s exact tests and chi-squared tests for larger tables. Patients where there was no recorded ASA grade were excluded from ASA analysis. Comparison between number of blood transfusions and was performed using a chi-squared test. Comparison between amount of perioperative blood transfusions and leak rate was performed with a chi-squared test for categories which had the same result as Poisson and ordered categories. Error rate was set at 0.05 and analysis was conducted using the SPSS software.

## Results

A total of 522 patients with colorectal cancer underwent colorectal cancer resection with a primary anastomosis (Table 1). Of these patients 442 (84.7%) patients required no blood transfusion and 80 (15.3%) patients required a blood transfusion. Of these patients, 19 (3.64%) procedures were complicated by an anastomotic leak. Patients who required a perioperative blood transfusion had a higher median age and lower pre-operative hemoglobin.

**Table 1:** Baseline characteristics

Variable	No transfusion	Transfusion	*p*-value
Number of patients	443 (84.5%)	79 (15.1%)	
Median age	71 (25–96)	73 (31–93)	0.005
Gender			
Male	237 (53.5%)	38 (48.1%)	0.3705
Female	206 (46.5%)	41 (51.9%)	
Mean BMI	27.9 (14.6–55.0)	27.0 (16–38.6)	0.57
Pre-operative hemoglobin (g/L)	124.87 (77–180)	106.62 (57–159)	< 0.001
Smoking status			
Active smoker	69 (16.31%)	8 (10.12%)	0.1613
Ex or non-smoker	354 (83.69%)	71 (89.88%)	
Type of anastomosis			
Hand sewn	136 (31.34%)	33 (41.77%)	0.0920
Stapled	298 (68.66%)	46 (58.23%)	
ASA			
1 and 2	180 (40.63%)	24 (30.78%)	0.1179
3, 4, and 5	260 (58.69%)	54 (68.35%)	
No grade	3 (0.68%)	1 (1.27%)	
T stage on histology			
0, 1, and 2	142 (32.05%)	9 (11.39%)	0.0019
3 and 4	301 (67.95%)	70 (88.61%)	
N stage on histology			
0	271 (62.01%)	39 (49.38%)	0.0469
1 and 2	166 (37.99%)	40 (50.62%)	
M stage on histology			
0	387 (87.36%)	68 (86.08%)	0.8954
1	56 (12.64%)	11 (13.92%)	

*BMI* body mass index, *ASA* American Society of Anesthesiologist, staging as per the TNM staging system

### Demographics

#### Blood Transfusions and Anastomotic Leak

Patients who had a blood transfusion after having a lower gastrointestinal anastomosis (Table 2) were significantly more likely to have anastomotic leak (*p* =  < 0.0001). The mean time to anastomotic leak diagnosis in patients who received a perioperative blood transfusion was 6.30 days, while the mean to leak diagnosis was 6.67 days for patients who did not receive a blood transfusion. There was no significant difference between these groups (*p* = 0.7386).

**Table 2:** Anastomotic leak prevalence after colorectal cancer resection between patients who received perioperative blood transfusion and patients who did not

	Anastomotic leak	No anastomotic leak	Rate of anastomotic leak	*p*-value
Transfusion	9	70	12.86%	< 0.0001
No Transfusion	10	433	2.31%	

#### Left and Right Colonic Procedures in Patients Receiving Perioperative Blood Transfusion

This increased rate of anastomotic leak was seen in patients who had a perioperative blood transfusion (Table 3) following surgery on the right colon (*p* = 0.02) and the left colon (*p* = 0.01). The difference in the rates of perioperative blood transfusion between patients who had right colonic versus left colonic surgery approached significance in this dataset (*p* = 0.06) with 17.87% of patients with right colonic surgery requiring a blood transfusion and 11.74% of patients undergoing left colonic surgery requiring a blood transfusion.

**Table 3:** The rate of perioperative transfusions in patients undergoing right and left colonic resection for colorectal cancer

		Number of patients	Number of anastomotic leaks	Rate of anastomotic leak	*p*-value
Right colonic surgery	Transfusion	51	5	9.80%	0.02
	No transfusion	240	5	2.08%	
Left colonic surgery	Transfusion	25	4	16%	0.01
	No transfusion	188	5	2.66%	

#### Types of Procedure and Anastomotic Leak

Of all the procedures performed requiring a lower gastrointestinal anastomosis, right hemicolectomy was the most common with 245 procedures performed across the time period (46.93% of all procedures). Other operations with a primary lower gastrointestinal anastomosis included in the study were extended right hemicolectomies (*n* = 50, 9.58% of all procedures), left hemicolectomy (*n* = 28, 5.36% of all procedures), sigmoid colectomy (*n* = 5, 0.96% of all procedures), total colectomy (*n* = 5, 0.96% of all procedures), subtotal colectomy (*n* = 8, 1.53% of all procedures), high anterior resection (*n* = 113, 21.64% of all procedures), low anterior resection (*n* = 37, 7.09% of all procedures), and transverse colectomy (*n* = 1, 0.19% of all procedures). There was no significant difference between rates of anastomotic leak between right colonic and left colonic surgery in this dataset (*p* = 0.64).

#### Quantity of Blood Transfusions and Rate of Anastomotic Leak

**Fig. 1:**
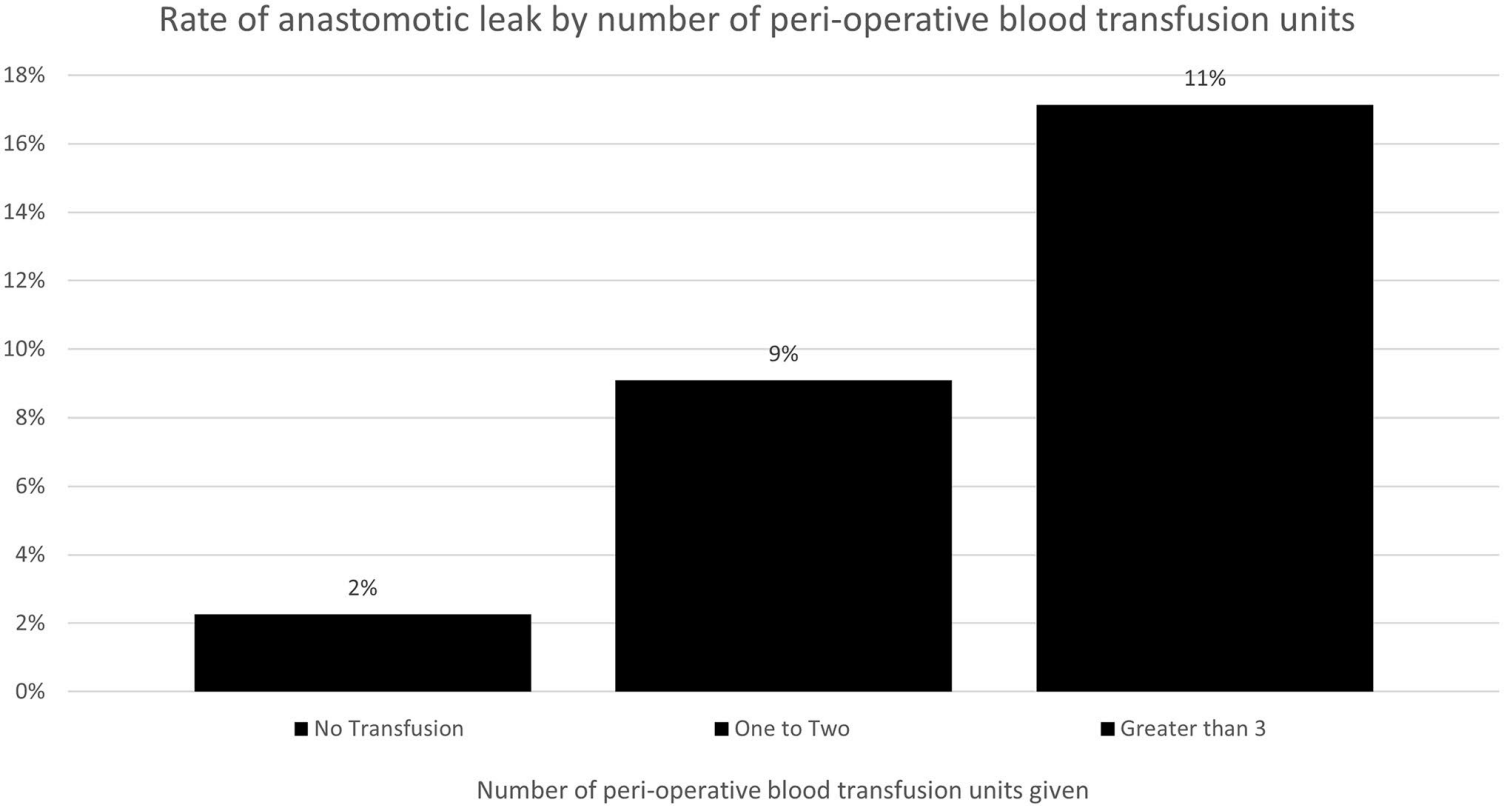
Rate of anastomotic leak after colorectal cancer resection by quantity of perioperative blood transfusions

Patients who received a greater quantity of units of blood transfusion prior to their diagnosis of anastomotic leak were more likely to develop an anastomotic leak (*p* < 0.001) (Fig. 1).

#### Timing of Blood Transfusion and Rate of Anastomotic Leak

In patients who received a blood transfusion, 23 had their first blood transfusion pre-operatively, 19 had their first blood transfusion intra-operatively, and 38 had their first blood transfusion post-operatively (Table 4). There was no difference in the incidence of anastomotic leak by timing of blood transfusion as the study did not have enough power to differentiate between the groups.

**Table 4:** Rate of anastomotic leak comparing patients who had pre-operative and intra-operative blood transfusions to patients who had post-operative blood transfusions

Peri-operative blood transfusion	Number of patients	Number of anastomotic leaks	Rate of anastomotic leak	*p*-value
Pre-op and intra-op transfusion	42	3	7.14%	0.22
Post-op transfusion	38	6	15.79%	

#### Blood Transfusion and Leak Management

Patients who had a peri-operative blood transfusion where just as likely to be managed with a procedure that resulted in a stoma as patients who did not have a peri-operative blood transfusion (*p* = 0.5637). Of the 19 patients diagnosed with an anastomotic leak, 12 were managed with surgery and formation of a stoma.

#### Blood Transfusions and Surgical Complications

Patients who had a blood transfusion were more likely have a Clavien-Dindo grade 3 or greater complication than those who did not have a blood transfusion after having a lower gastrointestinal anastomosis. As a blood transfusion is a Clavien-Dindo grade 2 complication, low grade complications were not included in the analysis (Table 5).

**Table 5:** Rates of Clavien-Dindo complications in patients who had a perioperative blood transfusion after resection for colorectal cancer with primary anastomosis

	Clavien-Dindo ≥ 3	Clavien-Dindo < 2	*p*-value
Transfusion	25	55	0.003
No transfusion	58	384	

#### Operative Urgency and Comorbidities and Perioperative Blood Transfusion

Patients who had an emergency or urgent procedures were more likely to have a perioperative blood transfusion (Table 6). Emergency procedures were defined as patients admitted from the emergency department or outpatient clinic who had their operation that admission, urgent was a procedure within 30 days of the diagnosis and elective was for operations delayed by greater than thirty days (usually for chemotherapy or radiotherapy prior). Respiratory comorbidities and diabetes mellitus were not associated with perioperative blood transfusion (Table 7). Patients with cardiac comorbidities approached statistical significance (*p* = 0.0647).

**Table 6:** Operative urgency, various comorbidities, and perioperative blood transfusion in patient undergoing resection of colorectal cancer with primary anastomosis

		Transfusion	No transfusion	*p*-value
Operative urgency	Delayed greater than 30 days	5 (6.0%)	78 (94.0%)	0.009
	Within 30 days	63 (14.0%)	323 (86.0%)	
	Emergency	12 (22.6%)	41 (77.4%)	
Comorbidities	Cardiac	58 (17.3%)	278 (82.7%)	0.0647
	No cardiac	22 (13.4%)	164 (86.6%)	
	Respiratory	25 (15.5%)	136 (84.5%)	0.8073
	No respiratory	55 (18.0%)	306 (82.0%)	
	Diabetes mellitus	13 (13.8%)	81 (86.2%)	0.5667
	No diabetes mellitus	67 (15.7%)	361 (84.3%)

**Table 7:** List of variables analyzed in cohort of patients undergoing colorectal cancer procedures with a primary anastomosis and their association with anastomotic leak

Variable	Anastomotic leak	No anastomotic leak	*p*-value
Number of patients	19 (3.64%)	503 (96.36%)	
Median age	72.3 (52–88)	68.6 (25–96)	0.0002
Gender			
Male	15 (78.95%)	259 (51.49%)	0.019
Female	4 (21.05%)	244 (48.51%)	
Mean BMI	29.5 (22.0–7.9)	27.7 (14.6–55)	0.0005
Pre-operative hemoglobin (g/L)	125.6 (93–159)	121.97 (57–180)	0.46
Smoking status			
Active smoker	4 (5.0%)	73 (95.0%)	0.43
Ex or non-smoker	15 (3.4%)	430 (96.6%)	
Type of anastomosis			
Hand sewn	8 (4.6%)	166 (95.4%)	0.5630
Stapled	11 (2.2%)	337 (97.8%)	
ASA			
1 and 2	2	201	0.0128
3, 4, and 5	16	298	
No grade	1	4	
T stage on histology			
0, 1, and 2	2	154	0.06
3 and 4	17	349	
N stage on histology			
0	6	288	0.025
1 and 2	13	213	
M stage on histology			
0	15	446	0.20
1	4	57	
Cardiac comorbidities			
Yes	16	319	0.11
No	3	183	
Respiratory comorbidities			
Yes	12	149	0.01
No	7	354	
Type 2 diabetes mellitus			
Yes	16	319	0.06
No	3	184	

#### Multivariate Analysis of Causes of Anastomotic Leak in Data

Analysis of other variables that could be contributing to anastomotic leak found an association with age of patient (*p* = 0.0002), gender (*p* = 0.019), BMI (*p* = 0.0005), ASA (*p* = 0.012), N stage on histology (*p* = 0.025), and respiratory comorbities (*p* = 0.01) (see Table 7).

## Discussion

In this study, perioperative blood transfusion was associated with an increased risk of clinically significant anastomotic leak in patients undergoing restorative colorectal cancer surgery. Patients who received higher volumes of perioperative blood transfusion were more likely to have an increased rate of anastomotic leak. The rate of anastomotic leak in our study in patients receiving a perioperative blood transfusion was 12.86% compared to 2.31%for patients who did not receive a perioperative blood transfusion. Other studies have reported associations between perioperative blood transfusion and lower gastrointestinal anastomotic leak [10, 17–21], however there is no uniform definition of anastomotic leak or perioperative blood transfusion in the literature [22]. This study also demonstrated an association of anastomotic leak (see Table 7) with age of patient (*p* = 0.0002), gender (*p* = 0.019), BMI (*p* = 0.0005), ASA (*p* = 0.012), N stage on histology (*p* = 0.025), and respiratory comorbities (*p* = 0.01), which have been shown to be associated with anastomotic leak in previous studies [1, 6, 14].

Different mechanisms have been proposed to explain why blood transfusion may be associated with anastomotic leak. It has been proposed that the use of intra-operative and post-operative blood transfusion may be a surrogate marker for operative difficulty and quality of the subsequent anastomosis [6, 23], however this mechanism alone would not explain why pre-operative blood transfusions are associated with anastomotic leak. Other explanations for anastomotic leak could be that requirement for blood transfusion may be a marker for pre-operative patient physiology.

A previous study has shown that blood exposure could be highly immunogenic in patients who had received a kidney transplant [24]. Another previous study has shown perioperative blood transfusion can delay wound healing after elective hip surgery [25]. Quantity of perioperative transfused units of blood did have an association with a higher rate of anastomotic leakage in this study.

Timing of the perioperative blood transfusion did not seem to make a statistically significant difference in terms of anastomotic leak in this dataset. 7.14% of patients with either a pre-operative or intra-operative blood transfusion suffered an anastomotic leak, compared to 15.79% of patients whose surgery suffered an anastomotic leak after a post-operative blood transfusion (*p* = 0.22). While not statistically significant in this study, it may be possible that post-operative blood transfusion increases the risk of anastomotic leak more than pre-operative or intra-operative blood transfusion. Additional study would be required to investigate this claim. Transfusions were not included if they were given after the anastomotic leak was diagnosed. Other studies have examined the role of intra-operative blood transfusion and found an association with anastomotic leak [14].

Procedures on both the right and left colon were associated with anastomotic leak if the patient had a perioperative blood transfusion. This is similar to other studies which have found an association between anastomotic leak in operations on the right colon [26] and the left colon [15]. In this study, patients who had a blood transfusion and a left sided colonic anastomosis where more much more likely to have an anastomotic leak that patients who had a blood transfusion and a right sided colonic anastomosis (16% vs 9.26%).

In this study, there were differences other that rate of anastomotic leak between patients who received a perioperative blood transfusion and those who did not. Patients who received a perioperative blood transfusion were more likely to have a lower pre-operative hemoglobin (*p* < 0.001) and have higher T and N stage disease (*p* = 0.004 and 0.049 respectively). This could be explained by higher stage local disease being more likely to result in blood loss into the bowel and causing pre-operative anemia [26]. Additionally, older patients were more likely to receive a peri-operative blood transfusion.

## Conclusion

Perioperative blood transfusions are associated with a significantly increased risk of an anastomotic leak following bowel resection with primary anastomosis for colorectal cancer. Surgeons who are operating on patients who require pre-operative and intraoperative blood transfusions should consider the heightened risk of post-operative anastomotic leak. Clinician’s index of suspicion of anastomotic leak should be heightened in patients who receive a perioperative blood transfusion after lower gastro-intestinal surgery, particularly if they have had multiple transfusions. Further studies should evaluate whether minimizing use of blood transfusions is associated with better patient outcomes.

## References

[1] Alves A, Panis Y, Trancart D, Regimbeau JM, Pocard M, Valleur P (2002). Factors associated with clinically significant anastomotic leakage after large bowel resection: multivariate analysis of 707 patients. World J Surg.

[2] Frye J, Bokey EL, Chapuis PH, Sinclair G, Dent OF (2009). Anastomotic leakage after resection of colorectal cancer generates prodigious use of hospital resources. Colorectal Dis.

[3] Mirnezami A, Mirnezami R, Chandrakumaran K, Sasapu K, Sagar P, Finan P (2001). Increased local recurrence and reduced survival from colorectal cancer following anastomotic leak: systematic review and meta-analysis. Ann Surg.

[4] McArdle CS, McMillan DC, Hole DJ (2005). Impact of anastomotic leakage on long-term survival of patients undergoing curative resection for colorectal cancer. Br J Surg.

[5] Hammond J, Lim S, Wan Y, Gao X, Patkar A (2014). The burden of gastrointestinal anastomotic leaks: an evaluation of clinical and economic outcomes. J Gastrointest Surg.

[6] Park JS, Huh JW, Park YA, et al. Risk factors of anastomotic leakage and long-term survival after colorectal surgery. Medicine (Baltimore). 2016;95:e2890. 10.1097/MD.0000000000002890. 10.1097/MD.0000000000002890 PMC4779025 26937928

[7] Bakker IS, Grossmann I, Henneman D, Havenga K, Wiggers T (2014). Risk factors for anastomotic leakage and leak-related mortality after colonic cancer surgery in a nationwide audit. Br J Surg.

[8] Foppa C, Ng SC, Montorsi M, Spinelli A (2020). Anastomotic leak in colorectal cancer patients New insights and perspectives. Eur J Surg Oncol.

[9] Chadi SA, Fingerhut A, Berho M (2016). Emerging trends in the etiology, prevention, and treatment of gastrointestinal anastomotic leakage. J Gastrointest Surg.

[10] McDermott FD, Heeney A, Kelly ME, Steele RJ, Carlson GL, Winter DC (2015). Systematic review of preoperative, intraoperative and postoperative risk factors for colorectal anastomotic leaks. Br J Surg.

[11] Ramphal W, Boeding JRE, Gobardhan PD (2018). Oncologic outcome and recurrence rate following anastomotic leakage after curative resection for colorectal cancer. Surg Oncol.

[12] Nesbakken A, Nygaard K, Lunde OC (2001). Outcome and late functional results after anastomotic leakage following mesorectal excision for rectal cancer. Br J Surg.

[13] Karliczek A, Harlaar NJ, Zeebregts CJ, Wiggers T, Baas PC, van Dam GM (2009). Surgeons lack predictive accuracy for anastomotic leakage in gastrointestinal surgery. Int J Colorectal Dis.

[14] Cortina CS, Alex GC, Vercillo KN (2019). Longer operative time and intraoperative blood transfusion are associated with postoperative anastomotic leak after lower gastrointestinal surgery. Am Surg.

[15] Makela JT, Kiviniemi H, Laitinen S (2003). Risk factors for anastomotic leakage after left-sided colorectal resection with rectal anastomosis. Dis Colon Rectum.

[16] Bruce J, Krukowski ZH, Al-Khairy G, Russell EM, Park KG (2001). Systematic review of the definition and measurement of anastomotic leak after gastrointestinal surgery. Br J Surg.

[17] Tartter PI (1988). Blood transfusion and infectious complications following colorectal cancer surgery. Br J Surg.

[18] Choi HK, Law WL, Ho JW (2006). Leakage after resection and intraperitoneal anastomosis for colorectal malignancy: analysis of risk factors. Dis Colon Rectum.

[19] Kang J, Kim H, Park H, Lee B, Lee KY. Risk factors and economic burden of postoperative anastomotic leakage related events in patients who underwent surgeries for colorectal cancer. PLoS One. 2002;17:e0267950. 10.1371/journal.pone.0267950. 10.1371/journal.pone.0267950 PMC9116683 35584082

[20] Boccola MA, Buettner PG, Rozen WM (2011). Risk factors and outcomes for anastomotic leakage in colorectal surgery: a single-institution analysis of 1576 patients. World J Surg.

[21] Leichtle SW, Mouawad NJ, Welch KB, Lampman RM, Cleary RK (2012). Risk factors for anastomotic leakage after colectomy. Dis Colon Rectum.

[22] Daniel VT, Alavi K, Davids JS, Harnsberger CR, Maykel JA (2021). Defining anastomotic leaks after colorectal surgery: results of a national survey. J Surg Res.

[23] Fjederholt KT, Svendsen LB, Mortensen FV (2017). Perioperative blood transfusions increases the risk of anastomotic leakage after surgery for GEJ-cancer. Am J Surg.

[24] Opelz G, Sengar DP, Mickey MR, Terasaki PI. Effect of blood transfusions on subsequent kidney transplants. Transplant Proc. 1973;5:253-259. 4572098

[25] Weber EWG, Slappendel R, Prins MH, van der Schaaf DB, Durieux ME, Strümper D (2005). Perioperative blood transfusions and delayed wound healing after hip replacement surgery: effects on duration of hospitalization. Anesth Analg.

[26] Frasson M, Granero-Castro P, Ramos Rodriguez JL (2016). Risk factors for anastomotic leak and postoperative morbidity and mortality after elective right colectomy for cancer: results from a prospective, multicentric study of 1102 patients. Int J Colorectal Dis.

